# Influence of Heart Rate and Change in Wavefront Direction through Pacing on Conduction Velocity and Voltage Amplitude in a Porcine Model: A High-Density Mapping Study

**DOI:** 10.3390/jpm14050473

**Published:** 2024-04-29

**Authors:** Theresa Isabelle Wilhelm, Thorsten Lewalter, Judith Reiser, Julia Werner, Andreas Keil, Tobias Oesterlein, Lukas Gleirscher, Klaus Tiemann, Clemens Jilek

**Affiliations:** 1 Peter-Osypka Heart Centre Munich, Internistisches Klinikum München Süd, 81379 Munich, Germanyklaus.tiemann@tum.de (K.T.); 2 Eye Center, Medical Center, Faculty of Medicine, University of Freiburg, 79106 Freiburg, Germany; 3 Medical Graduate Center, TUM School of Medicine and Health, Technical University of Munich, 81675 Munich, Germany; 4 Department of Medicine, University of Bonn, 53127 Bonn, Germany; 5 Center for Preclinical Research, TUM School of Medicine and Health, Technical University of Munich, 81675 Munich, Germany; judith.reiser@tum.de (J.R.);; 6 Boston Scientific Medizintechnik GmbH, 40468 Düsseldorf, Germany; 7 Department of Internal Medicine I, TUM School of Medicine and Health, Technical University of Munich, 81675 Munich, Germany

**Keywords:** conduction velocity, voltage amplitude, high-density-mapping, wavefront, pacing, heart rate

## Abstract

Background: Understanding the dynamics of conduction velocity (CV) and voltage amplitude (VA) is crucial in cardiac electrophysiology, particularly for substrate-based catheter ablations targeting slow conduction zones and low voltage areas. This study utilizes ultra-high-density mapping to investigate the impact of heart rate and pacing location on changes in the wavefront direction, CV, and VA of healthy pig hearts. Methods: We conducted in vivo electrophysiological studies on four healthy juvenile pigs, involving various pacing locations and heart rates. High-resolution electroanatomic mapping was performed during intrinsic normal sinus rhythm (NSR) and electrical pacing. The study encompassed detailed analyses at three levels: entire heart cavities, subregions, and localized 5-mm-diameter circular areas. Linear mixed-effects models were used to analyze the influence of heart rate and pacing location on CV and VA in different regions. Results: An increase in heart rate correlated with an increase in conduction velocity and a decrease in voltage amplitude. Pacing influenced conduction velocity and voltage amplitude. Pacing also influenced conduction velocity and voltage amplitude, with varying effects observed based on the pacing location within different heart cavities. Pacing from the right atrium (RA) decreased CV in all heart cavities. The overall CV and VA changes in the whole heart cavities were not uniformly reflected in all subregions and subregional CV and VA changes were not always reflected in the overall analysis. Overall, there was a notable variability in absolute CV and VA changes attributed to pacing. Conclusions: Heart rate and pacing location influence CV and VA within healthy juvenile pig hearts. Subregion analysis suggests that specific regions of the heart cavities are more susceptible to pacing. High-resolution mapping aids in detecting regional changes, emphasizing the substantial physiological variations in CV and VA.

## 1. Introduction

In cardiac electrophysiology, comprehending the dynamics of conduction velocity (CV) and voltage amplitude (VA) is imperative for advancing strategies in managing cardiac arrhythmias. Recent studies, such as the one by Kircher et al., have highlighted the effectiveness of individually tailored substrate modification. This approach demonstrated a higher arrhythmia-free survival rate compared to conventional linear ablation techniques in patients with atrial fibrillation [1]. Substrate mapping is employed in this context to identify areas such as local abnormal ventricular activities (LAVA) [2], zones of slow conduction [3], low voltage areas [4,5], and complex fractionated atrial electrograms (CFAE) [6]. The visibility of these crucial regions often depends on the strategy of pacing, [7,8,9,10,11,12], as conduction is typically faster along the cardiac fibers than it is across them [13,14]. To accurately interpret the changes induced by pacing, understanding the physiological variations in VC and VA during this process is essential.

Previous studies have established that CV and VA are not static properties but are influenced by heart rate and pacing conditions. The concept of CV restitution, where rate-dependent changes in CV typically decrease at higher frequencies, is well-documented [3,15,16,17]. This phenomenon is crucial in understanding the stability of re-entry circuits in cardiac arrhythmias.

In this study, we investigate the influence of heart rate and changes in wavefront direction caused by electrical pacing on cardiac CV and VA in vivo in healthy pigs. We compare electroanatomic high-density maps generated during various pacing conditions to those obtained during intrinsic normal sinus rhythm (NSR). This approach allows an in-depth analysis of pacing-induced wavefront-direction CV and VA changes at different levels, encompassing whole heart chambers, subregions, and localized areas of 5 mm diameter circles. Our aim is to discern how different factors affect CV and VA in a healthy cardiac porcine model and use this information to potentially contribute to the optimization of treatment strategies for cardiac arrhythmias.

## 2. Materials and Methods

Four juvenile healthy pigs (German Landrace × Pietrain; 31–41 kg (mean 36 kg), 3–4 months old) were used for this study, with two being female (pigs 3 and 4). The pigs were housed in groups under conventional hygienic conditions in an AAALAC-accredited animal facility. An acclimatization period of at least 7 days prior to the experiments was maintained. Electrophysiologic studies were conducted in vivo during intrinsic sinus rhythm and electrical stimulation from varying locations and with varying pacing rates. The animal trial was approved by the government of Upper Bavaria (ROB-55.2-2532.Vet_02-17-174, approved on 6 June 2018).

### 2.1. Anesthesia and Vascular Access

The animals were fasted for 12 h with unrestricted access to water prior to anesthesia. The pigs were intramuscularly sedated with ketamine (10–15 mg/kg), azaperone (2 mg/kg), and atropine (1 mg). Electrophysiologic studies were carried out under general anesthesia induced with 1% and maintained with 2% propofol i.v. and mechanical ventilation. Analgesia was provided through metamizole (40–50 mg/kg i.v.) and fentanyl boluses (0.001–0.01 mg/kg i.v.) every 20–30 min. Intraoperative activated clotting time was maintained above 300 sec with heparin. Venous access was gained by placing sheaths in the V. femoralis and jugularis. Electrocardiogram and blood pressure were continuously monitored. Catheter placement was guided by fluoroscopy, with the reference catheter in the right atrium and the mapping catheter transvenously inserted in the right heart, allowing access to the left heart via a transseptal approach. The pacing catheter was placed transvenously for the RA, RV, and LA pacing from the coronary sinus and arterially for the LV pacing. At the end of the experiments, the animals were sacrificed under general anesthesia with a pentobarbital overdose (40 mg/kg i.v.).

### 2.2. Thoracotomy

A left thoracotomy was performed in three of the four pigs to facilitate epicardial pacing alongside endocardial pacing. Additionally, a nerve block was created with a combined solution (10 mL) of 2% xylocaine and 0.75% bupivacaine. Atracurium (0.3–0.6 mg/kg i.v.) was given to relax the intercostal muscles before opening the thorax.

### 2.3. Electroanatomical Mapping and Pacing

Electroanatomic activation and voltage mapping was performed during the intrinsic sinus rhythm and with epi- and endocardial pacing from different sites, each maintaining a constant pacing rate of between 120 and 171 bpm. (Refer to Table 1 for recorded maps and the mean heart rate during each procedure.) Due to animal welfare considerations, mapping constellations were limited. Pacing was executed using the EPS320 Cardiac Stimulator (MicroPace Pty Ltd., Homebush West, Australia), and the ultra-high-density mapping system Rythmia (Boston Scientific Corp., Marlborough, MA, USA), along with its proprietary mapping catheter Intellamap Orion (Boston Scientific Corp.), which was utilized for electroanatomic mapping. The catheter, bidirectionally steerable and basket-shaped, featured 64 electrodes spaced 2.5 mm apart on eight splines [18].

### 2.4. Post-Processing in Rhythmia

**Figure 1:**
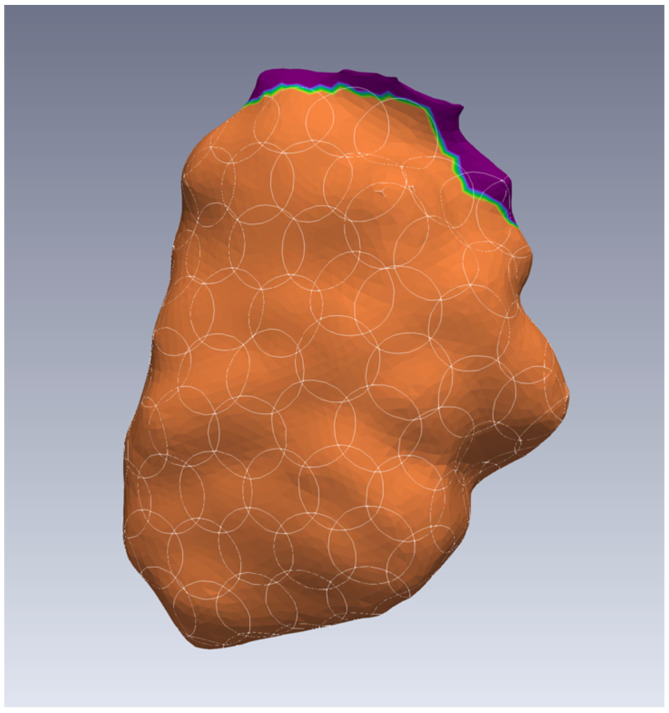
Example of the left ventricle (orange color) showing the circular areas (white outlines) for calculating local conduction velocities and voltage amplitudes in ParaView. The dark purple color indicates cutouts. In this case, it is the transition to the left atrium and the left ventricular outflow tract, where no circles have been placed.

Following the electrophysiological investigations, a careful manual review of the annotated beats was conducted to ensure their plausibility. If necessary, reannotation was performed within the Rhythmia software (Boston Scientific Corp., Marlborough, MA, USA; Version No. 3.0.0.4). The identification of transitions to different cardiac chambers, arteries, and veins was based on the electrogram’s morphological characteristics and amplitudes, and such transitions were denoted as cutouts (depicted in purple in Figure 1). For each mapping procedure, the average heart rate was estimated during the intrinsic sinus rhythm observation. 

### 2.5. Calculation of Local CV and VA

To calculate local the CVs and VAs, we employed a circle strategy, as outlined in our previous publication [19]. We covered the curved map surfaces with circular areas, using spherical filters with a radius of 5 mm within the software ParaView (v.5.8.0 & v.5.10.0, Kitware Inc., Clifton Park, NY, USA) [20,21]. Regions marked as cutouts were left blank, as shown in Figure 1, which uses a left ventricle as an example. To evaluate the different walls of each chamber separately, we created additional simulations, each containing just the septal, lateral, posterior, or anterior circular areas in the case of ventricles and the left atrium plus the superior of the atria.

For each circular area, we computed the mean VA and the CV. The CV was obtained by dividing the theoretical diameter of the circular area by the time difference between the first and last activation within the area. A custom-written Python script was used for the calculations and to export all values and metadata from ParaView to other software.

### 2.6. Statistical Analysis

The statistical analysis employed the R programming language v4.2.2 [22].

Given the repeated measurements with varying numbers of observations from four individuals, linear mixed-effects models (lme4) were used to assess heart rate, mapping location, and pacing location effects on CV and VA [23,24]. Heart rate and the combination of mapping and pacing location were treated as fixed effects. No interaction term was used, as we assumed that heart rate has the same effect on every heart chamber. Pig ID was selected as a random effect to account for possible inter-individual variations. The CV was log-transformed to achieve a normal distribution in the linear model.

Post hoc tests and comparisons were conducted using the lmerTest [25,26], and emmeans [27] packages. To adjust for multiple comparisons, Tukey’s HSD was used for all pairwise and Sidak for targeted comparisons.

Post hoc test results are expressed on the original scale. All values calculated by means of mixed-effects models are called estimates. All values are normalized to a heart rate of 90 or 145 bpm as indicated. Estimates are reported in the format (estimate ± standard error, *p*-value). Confidence intervals are 95% where explicitly indicated. Velocity values exceeding 6 m/s were considered outliers and disregarded in our calculations. A significance threshold of *p* < 0.05 was applied. Only unipolar signals are reported.

In addition to assessing overall differences through the mixed-effects model, we examined local pacing-induced VA and CV changes by assessing the absolute differences between the corresponding circles of the paced and intrinsic maps wherever the recordings allowed direct matching. The investigated parameters include:

The mean of absolute differences, to determine the size of the changes:

(1) $$mean\;of\;absolute\;differences=\frac{\sum \;\left|pace\;value-intrinsic\;value\right|}{Nr.\;of\;circles}$$

The absolute difference ratio, to determine the size of those changes relative to the mean of during sinus rhythm:

(2) $$absolute\;difference\;ratio=\frac{mean\;of\;absolute\;differences}{mean\;of\;intrinsic\;values}$$

Boxplots of the percentage differences of each circle relative to the mean intrinsic sinus rhythm within the map:

(3) $$relative\;change=\frac{pace\;value-intrinsic\;value}{mean\;intrinsic\;value}\times 100\%$$

The IQR of relative change to each intrinsic value:

(4) $$IQR\;of\;relative\;change=IQR\;of\;all\;\frac{pace\;value-intrinsic\;value}{intrinsic\;value}$$

## 3. Results

A total of 44 maps (recordings of individual heart cavities) were analyzed, each comprising 5593 ± 214 measurement points. The maps were covered with 134 ± 6 spheres, each covering an area of 77.8 mm^2^ ± 0.03 and including 104 ± 0.14 measurement points. The mapping resolution, therefore, was 1.3 points/mm^2^. We performed a linear mixed-effects analysis to discern the effects of heart rate, mapping location, and pacing location on conduction velocity (CV) and voltage amplitude (VA), accounting for inter-individual variance.

### 3.1. Influence of Heart Rate on CV and VA

We posited that heart rate has a consistent impact on all heart cavities, whether recorded during pacing or intrinsic sinus rhythm. Under this assumption, heart rate exhibited an overall significant effect on CV and VA (*p* < 0.001).

The estimated CV increased significantly with an increase in heart rate according to:

(5) $$CV=\left(chamber\;specific\;intercept\right)\times 1.004^{heart\;rate\;in\;bpm}$$

The estimated VA decreased by 0.012 mV ± 0.003 per 1 bpm increase in heart rate.

**Figure 2:**
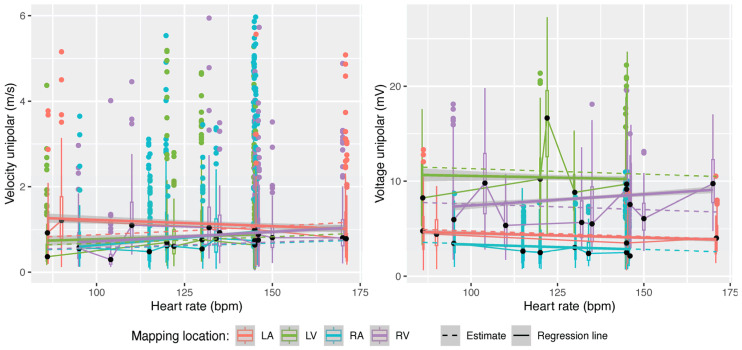
Influence of heart rate on unipolar CV (**left**) and VA (**right**). Measurements are shown as boxplots and their regression lines are in bold. Estimates from linear mixed-effects models are displayed as dashed lines.

Figure 2 illustrates the original measurements as boxplots and solid regression lines and the estimated values as dashed lines, organized according to the heart chambers tested. The estimated equations closely align with the trend of the measured values. Only one out of the four heart chambers in each category deviates from the estimated trend (CV in the LA and VA in RV) (Figure 2).

### 3.2. Influence of Pacing (Change in Wavefront Direction) on CV and VA

**Figure 3:**
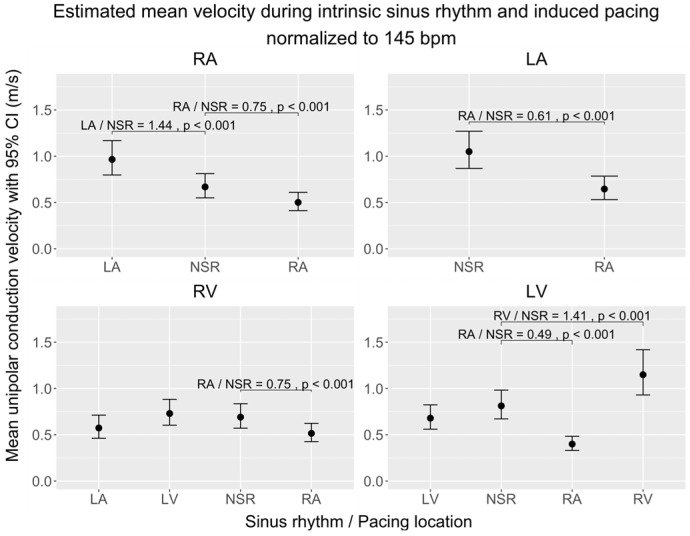
Comparison of estimated unipolar conduction velocities during sinus rhythm (NSR) and during induced pacing from various locations, normalized to 145 bpm, across the cardiac chambers (RA, LA, RV, LV) in a porcine model. The figure shows four subplots, each representing a cardiac chamber with potential pace modalities (intrinsic sinus rhythm or induced pacing from RA/LA/RV/LV), and pairwise comparisons between each pacing location and sinus rhythm. The 95% interval is given for each mean velocity. Brackets highlight significant contrast ratios (*p* < 0.05) to sinus rhythm, indicating divergence from a ratio of 1. Data were obtained using linear mixed-effects models and estimated marginal means. We conducted tests on the log scale and subsequently back-transformed the results for interpretation.

**Figure 4:**
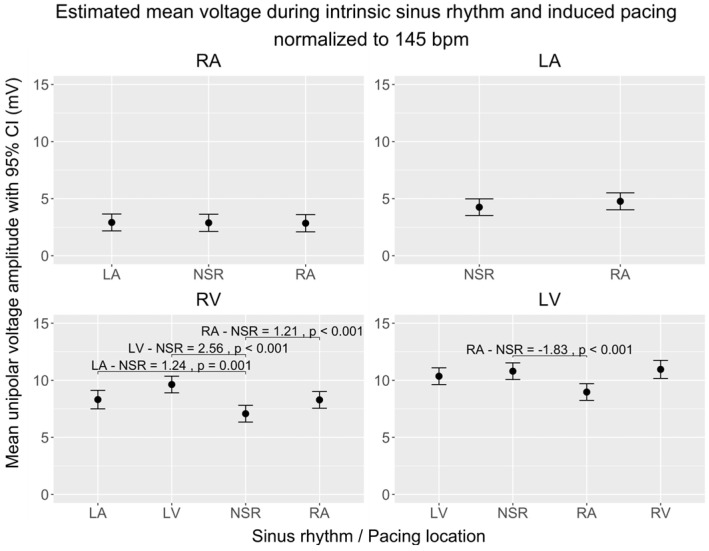
Comparison of estimated unipolar voltage amplitude means during sinus rhythm (NSR) and induced pacing from various locations, normalized to 145 bpm, across the cardiac chambers (RA, LA, RV, LV) in a porcine model. The figure shows four subplots, each representing a cardiac chamber with potential pace modalities (intrinsic sinus rhythm or induced pacing from RA/LA/RV/LV), and pairwise comparisons between each pacing location and sinus rhythm. The 95% interval is given for each mean velocity. Brackets highlight significant differences between mean voltage amplitude during sinus rhythm and during pacing from each location (Δ > 0 mV; *p* < 0.05). Data were obtained using linear mixed-effects models and estimated marginal means.

**Figure 5:**
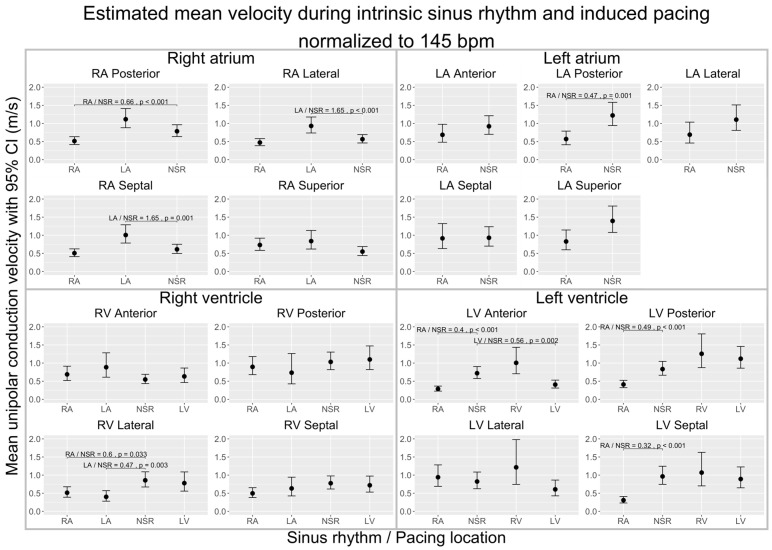
Estimated means of localized unipolar conduction velocity (m/s) in specific subregions of the heart depending on pacing location, or sinus rhythm (SR) normalized to a heart rate of 145 bpm in a porcine model. The 95% interval is given for each mean velocity. Significant contrast ratios (*p* < 0.05) of the unipolar CV between induced pacing and the intrinsic sinus rhythm within each wall of each heart chamber are indicated with brackets. Data were obtained using linear mixed-effects models and estimated marginal means, with tests conducted on the log scale and subsequent back-transformation of the results for interpretation.

**Figure 6:**
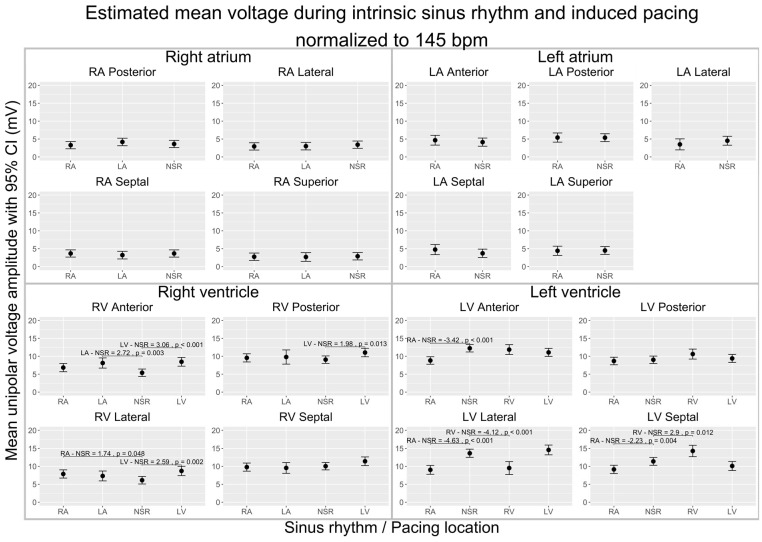
Estimated means of localized unipolar voltage amplitude (mV) in specific subregions of the heart depending on pacing location, or sinus rhythm (SR) normalized to a heart rate of 145 bpm in a porcine model. The 95% interval is given for each mean velocity. Significant differences (Δ > 0 mV; *p* < 0.05) of unipolar voltage amplitude during induced pacing from each tested location and intrinsic sinus rhythm within each wall of each heart chamber are indicated with brackets. Data were obtained using linear mixed-effects models and estimated marginal means.

To explore the effects of the wavefront direction on CV and VA, we compared maps of data gathered during pacing from different locations to maps of data gathered during sinus rhythm. To distinguish the effects of each pacing location from the influence of the heart rate, all the following estimates are normalized to a heart rate of 145 bpm. The overall CVs and VAs and the significant differences are displayed in Figure 3 (CV) and Figure 4 (VA). Table 2 and Table 3 present the absolute differences in local CVs and VAs and their distribution. Each chamber was subsequently subdivided into four to five subregions to allow more anatomically-precise observations of the effects of pacing. Estimates for these subregions are depicted in Figure 5 (CV) and Figure 6 (VA).

#### 3.2.1. Conduction Velocity

##### Overall Analysis

In the right atrium (RA), pacing from the RA decreased the overall CV (×0.75 ± 0.03, *p* < 0.001). Conversely, when pacing was initiated from the left atrium (LA), the CV in the RA increased (×1.44 ± 0.07, *p* < 0.001). In the left atrium (LA), pacing from the RA led to a decrease in the CV (×0.62 ± 0.05, *p* < 0.001). In the right ventricle (RV), pacing from the RA was associated with a decrease in the CV (×0.75 ± 0.05, *p* < 0.001). However, pacing from either the LA or the LV did not produce an effect on the CV in the RV. In the left ventricle (LV), pacing from the RA reduced the CV (×0.49 ± 0.03, *p* < 0.001), and pacing from the RV increased the CV (×1.4 ± 0.12, *p* < 0.001) (Figure 3).

##### Absolute Local Changes

**Figure 7:**
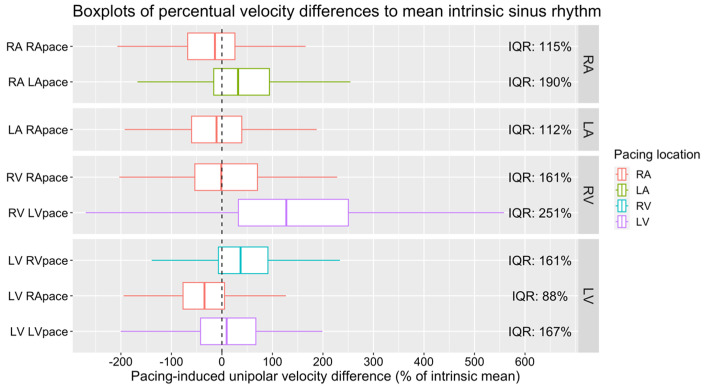
Distribution of relative CV change (in %) within each circle of the maps.

In addition to the estimated overall CVs, we calculated the mean of the absolute differences of the original measurements plus their IQR, the absolute difference ratio (relative to the mean NSR), and the IQR of the relative change within each circle (Table 2). These values show that with even a small change in the overall velocity, a large effect on local velocity due to pacing-induced change in the wavefront can be observed. The distribution of relative velocity changes within each circle of the maps is shown in Figure 7 as a boxplot with the IQR of the relative CV changes.

##### Regional Analysis

Within the RA subregions, pacing from the RA significantly reduced the CV in the posterior wall (×0.66 ± 0.06, *p* < 0.001). Conversely, pacing from the LA resulted in a significant increase in the CV in both the lateral (×1.65 ± 0.18, *p* < 0.001) and septal walls (×1.65 ± 0.20, *p* = 0.001). Pacing from the RA significantly decreased the CV in the posterior wall of the LA (×0.47 ± 0.09, *p* = 0.001). In the RV subregions, RA pacing led to a significant CV decline in the lateral wall (×0.60 ± 0.09, *p* = 0.03). Similarly, LA pacing also resulted in a decrease in the CV within the lateral wall (×0.47 ± 0.09, *p* = 0.003). However, pacing from the LV did not produce any significant alterations in the CV across the RV walls. Within the LV subregions, RA pacing was associated with a notable decrease in the CV across several walls: anterior (×0.40 ± 0.05, *p* < 0.001), posterior (×0.49 ± 0.06, *p* < 0.001), and septal (×0.32 ± 0.05, *p* < 0.001). LV pacing specifically reduced the CV in the anterior wall of the LV (×0.56 ± 0.08, *p* = 0.002). In contrast, RV pacing exhibited no significant impact on the CV within the RV walls (Figure 5).

##### Comparison of Overall and Regional Analysis

While RA pacing reduced the overall CV in all chambers, in the regional analysis this was only significant in the posterior wall of the RA and LA, the lateral wall of the RV, and the anterior, posterior, and septal wall of the LV. The overall CV increase in the RA during LA pacing was found in the lateral and septal wall of the RA. LA pacing did not change the overall CV in the RV, but in the lateral wall of the RV, a decrease in the CV was found. Pacing from the LV did not show a significant impact on the CV of the RV in either the overall or the regional analyses. In the LV, RV pacing increased the overall CV, but no wall differed significantly from sinus rhythm, and LV pacing did not have an overall effect but did decrease the CV in the anterior wall (Figure 3 and Figure 5).

#### 3.2.2. Voltage Amplitude

##### Overall Analysis

In the RA, pacing from the RA and LA did not significantly change the overall VA (Figure 4). The mean absolute difference of local VAs, however, was 1.61 mV ± 0.07, which is 49% of the intrinsic value for RA pacing (IQR of change 77%) and 1.34 mV ± 0.06, corresponding to 41% of the intrinsic value, for LA pacing (IQR of change 64%, Table 3). 

In the LA, pacing from the RA did not change the VA significantly (Figure 4). The absolute values differed by 1.94 mV ± 0.11 (45% of intrinsic value; IQR of change 79%, Table 3).

In the RV, pacing from the RA (+1.21 ± 0.22 mV, *p* < 0.001), LA (+1.236 ± 0.33 mV, *p* = 0.001), and LV (+2.55 ± 0.22 mV, *p* < 0.001) all increased the VA (Figure 4). The absolute values differed by 2.09 ± 0.14 (25% of intrinsic value; IQR of change 25%) and 3.24 ± 0.19 (32% of intrinsic value; IQR of change 54%) for RA and LV pacing (Table 3). For the RV map during LA pacing, a pairwise comparison of the absolute local VAs was impossible due to incongruences in the shapes of the recorded maps.

In the LV, pacing from the RA decreased the VA (−1.83 ± 0.22 mV, *p* < 0.001) and pacing from the RV and the LV did not change the VA significantly (Figure 4). The absolute values differed during RA pacing by 6.76 ± 0.32, corresponding to 50.99% of the intrinsic value (IQR of change 56%). The absolute values differed during RV pacing by 5.23 ± 0.38, which is 33% of the intrinsic value (IQR of change 33%), and during LV pacing by 4.51 ± 0.23, which corresponds to 24% of the intrinsic value (IQR of change 45%, Table 3).

##### Absolute Local Changes

**Figure 8:**
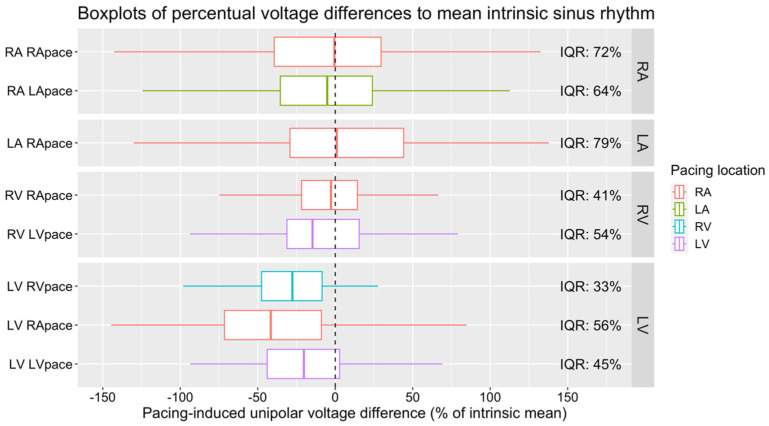
Distribution of relative VA change (in %) within each circle of the maps.

For the VA, as in the CV analysis, we calculated the mean of the absolute differences of the original measurements plus their IQR, the absolute difference ratio (relative to the mean NSR), and the IQR of the relative change within each circle (Table 3). Here, the absolute differences of the individual measurements were somewhat smaller than for the CV. It is still evident that even when only marginal voltage changes can be measured in the overall map, pacing induces a substantial impact on local voltage amplitude. To visually represent this, we illustrate the distribution of relative VA changes within each circle in Figure 8 using a boxplot format that highlights the interquartile range (IQR) of the relative VA shifts. 

##### Regional Analysis

Within the subregions of the RA, neither RA nor LA pacing produced any significant changes in the VA. This pattern remained consistent in the LA subregions, where RA pacing similarly did not influence the VA. Conversely, in the RV subregions, RA pacing was associated with an increase in the VA in the lateral wall (+1.74 ± 0.54, *p* = 0.048). Concurrently, LA pacing increased the VA in the anterior wall (+2.72 ± 0.69, *p* = 0.003). Pacing from the LV resulted in elevated VAs across several walls: the anterior (+3.06 ± 0.58, *p* < 0.001), the posterior (+1.98 ± 0.56, *p* = 0.013), and the lateral wall (+2.59 ± 0.64, *p* = 0.002) (Figure 6). 

##### Comparison of Overall and Regional Analysis

In the RA and LA neither the overall VA nor the voltage of any subregion differed from the intrinsic sinus rhythm during RA and LA pacing. In the RV, in the overall analysis RA, LA, and LV pacing all significantly changed the VA. Dividing the maps into four regions reveals that the main change happens in the lateral wall during RA pacing (+1.74 ± 0.54 *p* = 0.048), the anterior wall during LA pacing (+2.72 ± 0.69 *p* = 0.003), and the anterior, posterior and lateral walls during LV pacing (anterior +3.06 ± 0.58 *p* < 0.001, posterior +1.98 ± 0.56 *p* = 0.013, lateral +2.59 ± 0.64 *p* = 0.002). In the LV, an overall voltage change was only observed during RA pacing, visible in the anterior, lateral, and septal subregions (anterior −3.42 ± 0.44 *p* < 0.001, lateral −4.63 ± 0.65 *p* < 0.001, septal −2.23 ± 0.57 *p* = 0.004). In the subregions of the LV, we found additional changes in the lateral and septal walls during RV pacing; these changes are not visible in the overall VA (lateral −4.12 ± 0.94 *p* < 0.001, septal +2.90 ± 0.81 *p* = 0.012).

## 4. Discussion

### 4.1. Electroanatomical Mapping of All Heart Chambers

This study systematically analyses the influence of heart rate and pacing location on the local CVs and VAs in all four heart chambers and their subregions of healthy pigs in vivo utilizing an ultra-high-density mapping system. Iso et al. had a similar approach, but they studied only the LA under two different pacing frequencies and from three distinct atrial pacing sites in humans with atrial fibrillation [28].

In our study, unipolar signals were used, differing from the bipolar signals commonly used for generating ultra-high-density 3D voltage maps. Unipolar mapping’s efficacy in detecting changes in ventricular activation corresponding to histologically verified viable myocardial tissue within ischemic scar regions has been demonstrated [29]. The efficiency of unipolar mapping has also been demonstrated in delineating low voltage regions within the left atrium (LA).

The average mapping resolution in our analysis was 1.3 points/mm^2^, significantly higher than the resolutions in the published data. In a recent study characterizing left atrial slow conduction zones among patients with atrial fibrillations, the resolution was 0.2 points/mm^2^ [30].

### 4.2. Heart Rate Influence on CV

Contrary to the findings of previous studies, our findings indicate that there is a significant increase in overall CV connected to elevated heart rates. A study by Honarbakhsh et al. found stable CVs in the LAs of humans with atrial tachycardia at heart rates of up to 200 bpm, with a decrease observed at rates above 200 bpm and in low voltage areas (<0.5 mV) [3]. In monolayer cardiac tissue, a deceleration of conduction was observed at heart rates above 540 bpm [15]. Conversely, Weber et al. found a significant decrease in CV during in vivo pacing, ranging from 120 to 200 bpm, in the LAs of individuals with paroxysmal atrial fibrillation or focal right atrial tachycardia [17]. None of these studies described an augmenting effect of heart rate on CV. This inconsistency could be ascribed to the lower pacing frequencies employed in our study (max. 171 bpm) and the utilization of healthy, in vivo porcine hearts.

### 4.3. Heart Rate Influence on VA

The estimated VA demonstrated a significant dependence on heart rate. The effect of heart rate was calculated as a decrease of 0.012 mV ± 0.003 per 1 bpm increase in heart rate. Comparable to the findings of Iso et al., who studied LA voltage maps in patients with atrial fibrillation during RA pacing at 100 and 200 bpm, our results also indicate a heart rate-dependent change in VA [28]. In their study, the higher pacing frequency led to a 16% decrease in VAs from 4.00 mV at 100 bpm to 3.35 mV at 200 bpm. In our research, the VA showed a 21.8% decrease from 5.29 mV at 100 bpm to 4.13 mV at 200 bpm.

### 4.4. Effect of Pacing Location on Conduction Velocity

Altering the wavefront direction by pacing from different sites significantly affected the conduction velocity in some mapping-pacing combinations. RA pacing resulted in a reduced CV in all four chambers compared to the sinus rhythm, which is surprising since RA pacing is anatomically closest to physiologically intrinsic pacing via the sinus node. An increased CV was noted in the RA during LA pacing and in the LV during RV pacing. Similarly, Wong et al. investigated the effects of changing the wavefront direction on atria through distal coronary sinus pacing in patients with atrial fibrillation and patients with chronic atrial stretch, each compared to a reference group of patients with structurally normal hearts. It was shown that during pacing, the CV was slower, biatrial activation time was prolonged, the number and length of conduction block lines increased, the proportion of fractionated EGMs increased, and the VA decreased. Although these changes were more pronounced in patients with atrial fibrillation or chronic atrial stretch, they were also present in the reference group [31,32]. In our study, only healthy hearts without LAVA or slow conduction zones were analyzed, but as in Wong et al.’s study, an effect of changes in the wavefront direction on the conduction velocity was measurable.

In conclusion, we assert that the pacing location influences the CV in both the atria and the ventricles. Discrepancies in trial data regarding the effect of pacing on CV should be considered, as most trials were performed using non-ultra-high-density mapping systems, which may lack precision in analyzing the CV. A second reason for the discrepancy could be that pacing with diagnostic catheters is a relatively crude method; it remains uncertain how much tissue is captured by a stimulus. The decreased CV in the ventricles caused by pacing in the RA may be a hint that the direction of input in the AV node does influence the CV.

### 4.5. Effect of Pacing Location on Voltage Amplitude

Previous studies have shown that varying wavefronts can unveil discordant regions in infarct hearts, identified as areas of low voltage during one wavefront and not during another [8,12]. We investigated whether changing the wavefront direction also has an impact on the voltage amplitudes within healthy hearts. In our measurements, we could not detect any overall effect of pacing on the voltage amplitudes within the atria. In comparison, in a study by Iso et al. in patients with atrial fibrillation, LA bipolar VAs differed significantly between pacing from the high right atrium, proximal coronary sinus, and distal coronary sinus [28]. Within the RV, in our study, RA, LA, and LV pacing all increased VA. We could not find any comparable data about the RV.

Most previous studies focused on the LV, where we found a lowering effect of RA pacing and no effect of RV or LV pacing. Our result for RV pacing in the LV is comparable to a study by Nguyên et al. on patients with left bundle branch blocks, where RV pacing did not change the overall unipolar VA in the epicardial LV [11]. Similarly to our study, the absolute values varied considerably. A similar variability of unipolar VAs in the LV with RV pacing was seen in the porcine myocardial infarction model of Amorós-Figueras et al. RV pacing resulted in a change concerning a fixed threshold voltage in 10.4% of all studied sites. The IQR of change was 32.2%, comparable to 34% in our study [33]. Going further than our study, in which only pacing versus the sinus rhythm was tested, Brunckhorst et al. compared LVs during atrial and ventricular pacing in patients with myocardial infarction and ventricular tachycardia and found a strong overall correlation. Nevertheless, changing the activation sequence from atrial to ventricular pacing resulted in a >50% change in the VA in 28% of all sites [34].

### 4.6. Regional Differences in Electrophysiological Responses

An essential aspect of our study was the subdivision of each heart chamber into several subregions, enabling a more detailed analysis of the effects of pacing. This approach revealed that overall changes in the CV and VA are not uniformly reflected in all subregions of a heart chamber. There were cases where an overall change was observed only in specific walls of a chamber (e.g., the observed overall increase in CV in the RA during LA pacing is predominantly due to an increase in CV within the lateral and septal walls of the RA). There were also instances where a change was visible in individual walls but not in the overall map (e.g., significant VA changes during RV pacing were seen in the lateral and septal wall of the LV but were not visible in the overall LV analysis that comprises all subregions). This suggests that certain regions are more susceptible to electrophysiologic changes due to pacing. High-resolution mapping, therefore, has the potential to reveal hidden changes and identify the exact region responsible for conduction changes, thereby revealing critical ablation targets, as suggested by Seifert et al. and Glashan et al. [29,35]. However, one should be careful not to misinterpret the physiological variabilities, which are also reflected in the strong variability of the absolute differences.

### 4.7. Limitations

The primary limitation of this study is its small sample size, involving only four pigs, a constraint primarily due to considerations of animal welfare. Although random effects were included to account for individual variability, this small cohort limits the generalizability of the findings. Furthermore, the use of healthy porcine hearts as a model, while beneficial due to their physiological similarity to human hearts, may not fully represent the complexities of human cardiac pathologies [36,37]. This raises concerns about the direct transferability of the results to human conditions, particularly in the context of diseased hearts.

Another limitation of ultra-high-density mapping systems is that amplitudes are always the results of the sum vector of electrical capacity. Therefore, changes in CVs and VAs may be overinterpreted.

We used unipolar signal mapping, which is standard in high-density mapping procedures. One limitation of unipolar signals is an increased susceptibility to far-field signals in comparison to bipolar signals. Taking into account the advantages of unipolar versus bipolar signal mapping, unipolar signals were our choice because the focus of the analysis was on conduction velocity and voltage amplitude. Both parameters are affected by wavefront propagation dynamics when using bipolar signal mapping [38,39].

Mapping was performed under general anesthesia. The time required to prepare the sheaths was used to establish a stable level of anesthesia depth, which was maintained during the whole mapping process. Nevertheless, electrophysiological parameters may have been influenced by anesthesia or changes in the pigs’ stress levels, which is a limitation of the study.

One methodological limitation is the approach used to calculate the conduction velocities. The calculation is based on the time difference between the first and last excitation within a small circular area. This could potentially lead to misinterpretations in cases of colliding wavefronts, although such occurrences are expected to be rare in healthy hearts. Additionally, the sequential mapping of different heart chambers and segments introduces the possibility of time-dependent effects on the measured velocities and voltages, despite efforts to minimize these influences. The heart rate estimation, conducted over approximately five minutes, might also carry some inaccuracies, and the assumption of a linear relationship in the mixed-effects model, while providing a reasonable approximation, may not capture all of the complex interactions in cardiac electrophysiology.

### 4.8. Comparison with Research Results of Diseased Human Hearts

This study is in line with the questions of daily clinical practice surrounding how to map arrhythmias.

With regard to ventricular arrhythmias, Martin et al. showed that alternate activation wavefronts running perpendicular to the isthmus site may be more sensitive tools to use in identifying arrhythmogenic substrate and critical sites for reentries [10]. In our study, we showed that pacing sites significantly impact conduction velocity and voltage amplitude even in healthy hearts.

Looking at patients with atrial stretch of or atrial fibrillation, pacing via the coronary sinus resulted in a greater slowing of conduction velocities and a prolongation of biatrial activation time [32]. Moreover, complex fractionated atrial electrograms depend on the direction and rate of activation and are of a functional nature [9]. Even in our healthy pig hearts, we could observe differences in conduction velocities depending on the pacing site. Therefore, the activation front seems not to be relevant in either diseased or healthy myocardia.

## 5. Conclusions

In healthy pig hearts, an elevated heart rate correlates with an increase in conduction velocity and a decrease in voltage amplitude. Additionally, changes in the wavefront direction were found to significantly impact conduction velocity and voltage amplitude. Overall, there was considerable variability in CV and VA changes due to pacing within the cavities of healthy hearts. Pacing in the RA and LA decreased the CV in the other atrium. RA pacing had a slowing effect on the CV in both ventricles, whereas LA pacing had no effect. In addition, the analysis provides a detailed description of regional changes of the CV and VA in all four heart chambers.

The application of high-resolution mapping proved effective in identifying subtle changes in cardiac electrophysiology, highlighting its potential utility in detailed cardiac studies and guiding therapeutic interventions.

## Figures and Tables

**Table 1:** Mapping protocol: Mean heart rate during the performed mapping procedures. Columns represent the location of electric stimulation. Rows represent the mapping location.

		Sinus Rhythm/Pacing Location				
		Sinus Rhythm	RA	LA	RV	LV
**Mapping location**	**RA**	115, 115, 120, 134, 130, 95, 145	145epi, 145epi, 145epi, 120endo, 146endo	145epi, 145epi, 145epi		
	**LA**	86, 90, 145, 171	145endo, 171endo			
	**RV**	104, 95, 135, 150, 110, 132	145endo, 145epi, 146endo	145epi		145endo, 170endo, 146endo
	**LV**	122, 130, 120, 86	145epi, 145epi, 145epi		145epi	145epi, 145

**Table 2:** Absolute differences in local CV (in m/s) and their distribution during pacing and intrinsic sinus rhythm.

Mapping Location	Pacing Location	Mean of Absolute Differences (m/s)	IQR of Abs. Differences	Absolute Difference Ratio	IQR of Relative Change
RA	RA	0.61	0.62	0.72	115%
RA	LA	0.74	0.82	0.88	190%
LA	RA	0.73	0.74	0.68	112%
RV	RA	0.66	0.79	0.91	161%
RV	LV	0.62	0.73	1.76	251%
LV	RA	0.76	0.67	0.84	88%
LV	RV	0.63	0.60	0.84	161%
LV	LV	0.75	0.80	0.84	167%

**Table 3:** Absolute differences in local VA (in mV) and their distribution during pacing and intrinsic sinus rhythm.

Mapping Location	Pacing Location	Mean of Absolute Differences (mV)	IQR of Abs. Differences	Absolute Difference Ratio	IQR of Relative Change
RA	RA	1.43	1.58	0.47	72%
RA	LA	1.34	1.36	0.41	64%
LA	RA	1.94	2.01	0.45	79%
RV	RA	2.09	2.36	0.25	41%
RV	LV	3.24	2.99	0.32	54%
LV	RA	6.76	8.01	0.51	56%
LV	RV	5.23	5.98	0.33	33%
LV	LV	4.51	5.07	0.34	45%

## Data Availability

The data presented in this study are available on request from the corresponding author.
